# Retraction: Shape-specific silver nanoparticles prepared by microwave-assisted green synthesis using pomegranate juice for bacterial inactivation and removal

**DOI:** 10.1039/c8ra90094a

**Published:** 2018-12-03

**Authors:** Andrew Shore

**Affiliations:** Royal Society of Chemistry Thomas Graham House, Science Park, Milton Road Cambridge CB4 0WF UK advances-rsc@rsc.org

## Abstract

Retraction of ‘Shape-specific silver nanoparticles prepared by microwave-assisted green synthesis using pomegranate juice for bacterial inactivation and removal’ by Ekta Roy *et al.*, *RSC Adv.*, 2015, **5**, 95433–95442.

The Royal Society of Chemistry hereby wholly retracts this *RSC Advances* article due to concerns with the reliability of the data in the published article.

Identical photographs have been used to represent disk diffusion tests for different samples in Fig. 4.

The killing kinetics data in Fig. 5 illustrate duplication of data, which were reported as corresponding to different bacterial strains.

Identical confocal fluorescence images have been used to represent different samples in Fig. 6. The image has also been used in another publication.^1^

The bacterial growth curves in Fig. S3 illustrate duplication of data, which were reported as different bacterial strains. The growth curves duplicate data presented in other publications.^2,3^

Given the number and significance of the concerns, the validity of the data and, therefore, the conclusions presented in this paper are no longer reliable.

The Royal Society of Chemistry apologises for the fact that these concerns were not identified during the peer review process.

Santanu Patra, Rashmi Madhuri and Prashant K. Sharma oppose the retraction. Ekta Roy and Shubham Saha were contacted, but did not respond.

Signed: Andrew Shore, Executive Editor, *RSC Advances*.

Date: 23^rd^ November 2018.

## Supplementary Material
